# Toxic Effects of the Trap Crop *Solanum sisymbriifolium* on the Hatch and Viability of *Globodera pallida*

**DOI:** 10.2478/jofnem-2024-0027

**Published:** 2024-08-08

**Authors:** Lindsay Schulz, Inna Popova, Louise-Marie Dandurand

**Affiliations:** Department of Entomology, Plant Pathology, and Nematology, University of Idaho, Moscow, ID 83844; Department of Soil Science, University of Wisconsin-Madison, Madison, WI 53706

**Keywords:** *Globodera pallida*, nematode management, potato cyst nematode, *Solanum sisymbriifolium*, toxicity

## Abstract

*Globodera pallida*, the pale cyst nematode, is a quarantined potato pest first found in Idaho in 2006. The containment and eradication of this economically devastating pest has been the focus of control since its discovery. *Globodera pallida* survives for 30+ years in soil and can cause up to 80% yield loss in susceptible potato varieties. Soil fumigants have been key to eradication efforts but many have been banned. Therefore, new control methods are needed. *Solanum sisymbriifolium* induces hatching but limits *G. pallida* reproduction and can be used as an alternative control measure. However, as *S. sisymbriifolium* has little economic value as a crop and its seeds are largely unavailable, it has not been widely adopted by potato producers. There is evidence that this plant kills the nematode by producing toxins, although this is poorly understood. Liquid–liquid extraction of *S. sisymbriifolium* leaf and stem tissues by hexane and 1-butanol reduced hatch by 49.5%, and 68.3%, respectively, compared with the potato root diffusate control. Many chemicals may be responsible for this toxic effect, including steroidal glycoalkaloids produced by plants in the Solanaceae family. The discovery of novel chemistries for nematicide development would be valuable for potato cyst nematode control.

The obligate sedentary endoparasite of potato, *Globodera pallida* (Stone, 1973) Behrens 1975, was first reported in 2006 in Idaho potato growing regions of the United States (Hafez et al., 2007). Found in 55 countries, *G. pallida* is a threat to global food security as it can decrease the yield of susceptible potato up to 80% (CABI, 2018; Contina et al., 2019). The United States Department of Agriculture – Animal and Plant Health Inspection Services (USDA-APHIS) implemented a quarantine on this pest, as well as an eradication program to eliminate *G. pallida* from infested fields and prevent its movement via soil, plant material, and farm equipment (USDA-APHIS, 2009). Currently, 1,433 ha of potato fields have been infested with *G. pallida* in Idaho and 1,225 ha of associated fields that have been potentially exposed are regulated (USDA - APHIS, 2023). Annually, the Idaho economy loses $25 million due to the inability of farmers to plant potatoes in these fields, who instead plant less valuable crops (Koirala et al., 2020).

The trap crop, *Solanum sisymbriifolium,* induces the hatching of *G. pallida* but does not allow reproduction (Scholte, 2000a; Kooliyottil et al., 2016). In one growing season, *S. sisymbriifolium* can reduce *G. pallida* populations by 50%–80% (Scholte, 2000b; Scholte and Vos, 2000). When in rotation with a susceptible potato, the reproduction of *G. pallida* was reduced by 99% by *S. sisymbriifolium* compared with potato (Dandurand and Knudsen, 2016). *Solanum sisymbriifolium* contains many biologically active secondary metabolites, including glycoalkaloids, flavonoids, and steroids/ sterols (Ferro et al., 2005; Ibarrola et al., 2006; Chauhan et al., 2011). *Solanum sisymbriifolium* active metabolites have been shown to have antimicrobial, antioxidant, anti-inflammatory, analgesic, anti-diarrheal, and anti-diabetic activity (More, 2019). Although metabolically active, the activity of these metabolites against plant parasitic nematodes remains unclear. Specifically, the stem/leaf tissue of *S. sisymbriifolium*, contains approximately 68 mg/g of total alkaloids, 30 mg/g of total flavonoids, and 24 mg/g of total tannins in dry weight plant material (Gupta et al., 2014). The glycoalkaloid content of *S. sisymbriifolium* has been analyzed and multiple glycoalkaloids in the stem, leaf, and root tissue were reported (Popova et al., 2022). Stem tissue of *S. sisymbriifolium* was found to contain hydroxy-solamargine, solamargine isomer, sycophantine, solanigroside, hydroxy-sycophantine, malonyl-solamargine, hydroxy-solamargine, anguivioside XI, and lyconoside II (Popova et al., 2022). Leaf tissue contained hydroxy-solamargine, malonyl-solanandaine, sycophantine, hydroxy-sycophantine, hydroxy-solamargine, and lyconoside II (Popova et al., 2022). Solamarine, hydroxy-sycophantine, hydroxy-solamargine, anguivioside XI, lyconoside II, and solanigroside were found in the root tissue of *S. sisymbriifolium* (Popova et al., 2022). The nematicidal properties of these secondary metabolites from *S. sisymbriifolium* are largely uncharacterized.

Synthesized secondary metabolites are important in plant stress and defense responses (Hartmann, 1991; Schäfer and Wink, 2009). These secondary metabolites are the first line of chemical plant defenses that pathogens must overcome for successful infection. Many *Solanum* spp., including nightshades, potato, eggplant, and tomato, contain defensive secondary metabolites (Hartmann, 1991; Isah, 2019; Popova et al., 2022). This study will focus on the effects of secondary metabolites from *S. sisymbriifolium* on *G. pallida*. Flavonoids, a group of secondary metabolites found in *S. sisymbriifolium*, are chemically diverse and include anthocyanidins, flavonols, chalcones, flavanones, dihydroflavonols, and dihydrochalcones (Treutter, 2005). Steroids are another group of secondary metabolites and can be split into two subgroups: those that have plant physiological roles and those that have allelochemical properties (Dinan et al., 2001). Allelochemical substances include brassinosteroids, bufadienolides, cardenolides, cucurbitacins, ecdysteroids, steroidal alkaloids, steroidal saponins, glucosides, and glycosides (Dinan et al., 2001; Gajger and Dar, 2021).

Glycoalkaloids, a group of metabolites that are in a class of nitrogen-containing steroidal glycosides, are not essential for plant growth or function, but are known to have concentration-dependent toxicity to insects. Glycoalkaloids increase the mortality rate of the Colorado potato beetle, *Leptinotarsa decemlineata*, and the potato leafhopper, *Empoasca fabae* (Cantelo et al., 1987; Sanford et al., 1995; Sanford et al., 1996; Yencho et al., 2000). Additionally, glycoalkaloids are fatally toxic to *Biomphalaria glabrata* and *Lymnea cubensis* (Alzérreca and Hart, 1982; Bekkouche et al., 2000). Pure glycoalkaloids have been shown to reduce the hatch of *G. pallida* by 99% compared with the control (Pillai and Dandurand, 2021).

A gradient of solvents from non-polar to polar is required for the effective extraction of all secondary metabolites found in *S. sisymbriifolium* (Dinan et al., 2001) A non-polar solvent like hexane will extract isoflavones, non-polar lipids, and sterols (Dinan et al., 2001). A moderately non-polar solvent like dichloromethane will extract certain flavonols, flavonones, brassinosteroids, cucurbitacins, and ecdysteroids (Tzanova et al., 2020). Moderately polar solvents like ethyl acetate will extract cardenolides and flavones. Polar solvents like water saturated 1-butanol will extract anthocyanins, glucosides, glycosides, glycoalkaloids, and steroidal saponins (Dinan et al., 2001; Sánchez-Maldonado et al., 2014).

Information about the toxic properties of secondary metabolites from *S. sisymbriifolium* to nematodes has not yet been established. The current study assesses the nematicidal properties of extracts from *S. sisymbriifolium* against *G. pallida*, via hatch and viability assays. The potential discovery of secondary metabolites from *S. sisymbriifolium* for the development of a nematicide would be a valuable achievement for potato cyst nematode control.

## Materials and Methods

### Rearing of *Globodera pallida*

Infested fields in Shelley, Idaho, were the original source of *Globodera pallida* for these experiments. Morphological and molecular methods were used to identify the Idaho *G. pallida* population (Skantar et al., 2007). *Globodera pallida* cysts were reared on the potato cultivar ‘Russet Burbank’ under greenhouse conditions (18°C; 16:8 light:dark photoperiod) for 16 wk according to methods described previously (Dandurand and Knudsen, 2016). Before experimental use, extracted cysts were stored at 4°C for a minimum of 16 wk.

### Growth of *Solanum sisymbriifolium*

*Solanum sisymbriifolium* seeds were planted in trays of promix BX (Premier Horticulture Inc., Quakertown, PA), and after germination, grown for 4 wk under greenhouse conditions (18°C; 16:8 light:dark photoperiod). Four-wk-old seedlings were transplanted into 15.24 cm clay pots containing a 3:1 ratio of sterilized Lane Mountain 20/30 industrial silica sand to silt loam soil Mission-series (University of Idaho – Sandpoint Organic Agriculture Center, Sandpoint, ID). Seedlings were maintained under greenhouse conditions at 60% relative humidity for 4, 6, or 8 wk, with pots watered daily. At planting, a slow-release fertilizer, Osmocote (The Scotts Company, Marysville, OH), was applied. An all-purpose 20-20-20 water soluble fertilizer was applied to the plants weekly (Jack’s Classic All Purpose, JR Peters Inc., Allentown, PA). To prevent thrips, weekly applications of Bioworks SuffOil-X horticultural oil (Bioworks, Victor, NY) were applied.

### Extraction of *Solanum sisymbriifolium*

Four-, six-, and eight-wk-old *S. sisymbriifolium* plants were terminated by clipping the plant’s stem at soil level, removing the aerial part of the plant and freezing it immediately in liquid nitrogen. Roots were removed from the soil, rinsed and flash-frozen immediately in liquid nitrogen. Plants were then placed in a FreeZone small tray freeze drier (LABCONCO, Kansas City, MO) for 60 hr the same day they were terminated. Once freeze-dried, plants were placed in an airtight Ziplock bag and stored in a −20°C freezer. Freeze-dried plants were ground with a cyclone sample mill (Udy Analyzer Company, Boulder, CO) plant material grinder, keeping the aerial part of the plant separate from the roots of the plant. Plant material was then extracted using a liquid-liquid extraction technique (Mazzola et al., 2008) with a rotavapor R-300 (Buchi, Switzerland). The four consequent solvents used were high-performance liquid chromatography (HPLC) grade hexane, dichloromethane, ethyl acetate, and 1-butanol. This created a total of eight extracts, with each extract being used either in an undiluted form or diluted 1:2.5, 1:5, or 1:10 for hatch and viability analysis. Extracts were used within 6 months and all extracts used in these experiments came from the same extraction batch.

### Potato Root Diffusate and Bare Soil Diffusate

Russet Burbank potato plants were cut and placed in tissue culture jars and grown for 4 wk. Plants were then transferred into 15.24 cm clay pots in 2:1 sterilized Lane Mountain 20/30 industrial silica sand to silt loam soil Mission-series (University of Idaho – Sandpoint Organic Agriculture Center, Sandpoint, ID). At the same time, pots were filled with soil and no plants, but treated the same as pots with plants in them. After 6 wk of growth under greenhouse conditions (18°C; 16:8 light:dark photoperiod), the pots were hydrated with 200-ml reverse osmosis deionized water (Millipore MilliQ, Darmstadt, Germany). After 2 hr, 200 ml of reverse osmosis deionized water was poured into the pots with and without plants and the flowthrough was collected. The flowthrough was filtered through a 0.45-μm-pore filter (Corning disposable vacuum filter, Corning, NY), which removed soil particles and debris. Then, the diffusate was filtered through a 0.22-μm-pore filter to sterilize the diffusate of microbes (Corning disposable vacuum filter, Corning, NY). The diffusate from potato plants and bare soil was then frozen at −20°C for up to 6 months until use in hatch and viability assays.

### Hatching assays

Extracts were tested for nematicidal properties via in vitro hatch assays. Cysts of *G. pallida* were surface sterilized in a final concentration of 0.3% hypochlorous bleach for 5 min followed by a total of five rinses with sterile distilled water (Nour et al., 2003). Hatch assays were performed in 96 well plates. One hundred microliters of potato root diffusate (PRD) was pipetted into each well, then 100 μl of the correct extract was pipetted into each well. Extracts were dissolved in sterile deionized water via sonication with a Q125 sonicator (QSonica, LLC, Newtown, CT). One surface sterilized cyst was placed in each well. Cysts were incubated at 18°C (Low Temperature BOD Refrigerated Incubator, Thermo Fisher Scientific, Marietta, OH) for 1 wk, emerged J2s were counted using a model no. DMi1 steromicroscope (Leica Microsystems, Wetzlar, Germany), and cysts were transferred to a fresh 96-well plate containing PRD only. Cysts were incubated at 18°C (Low Temperature BOD Refrigerated Incubator, Thermo Fisher Scientific, Marietta, OH) for an additional 2 wk and emerged J2s were counted. Cysts were crushed and the remaining encysted eggs with J2s were counted. There were four replicates per treatment for all experiments, with each replicate consisting of an average of two wells. The experiment was repeated. Hatch percentage was calculated as follows:

$$\left(\frac{\text{number}\;\text{J}2\text{s}\;\text{hatched}}{\text{initial}\;\text{J}2\;\text{in}\;\text{eggs}\;\text{number}}\right)\times 100$$

### Viability assays

Extracts were tested for nematicidal properties via in vitro viability assays. Cysts of *G. pallida* were surface sterilized in 0.3% hypochlorous bleach for 5 min followed by a total of five rinses with sterile distilled water (Nour et al., 2003). Viability assays were performed in 96-well plates. One hundred microliters of PRD was pipetted into each well, then 100 μl of the correct extract was sonicated with a Q125 sonicator (QSonica, LLC, Newtown, CT) into sterile deionized water. This was pipetted into each well. One surface sterilized cyst was placed in each well. Cysts were incubated at 18°C for 1 wk. The viability of *G. pallida* eggs after exposure was assessed using the acridine orange staining method (Pillai and Dandurand, 2019), in which eggs are stained with acridine orange to see if they uptake the stain. Eggs that are stained and fluoresce are not viable. Cysts were removed from the PRD/extract solution and placed in fresh sterile water, then crushed. Acridine orange was applied and after 4 hr was visualized under a Leica DMi8 fluorescent microscope (Leica microsystems CMS GmbH, Wetzlar, Germany), which was equipped with a metal halide light (Lumen 200 Fluorescence Illumination Systems, Prior Scientific Inc., Rockland, MA). The total number of fluorescing eggs was counted as well as the total number of eggs in each well. The fluorescing stain can move through the eggshell membrane and cause the egg to fluoresce, meaning that the permeability of the eggshell membrane is lower for those eggs, indicating that they are no longer viable to hatch. There were four replicates per treatment and each replicate consisted of an average of two wells. The experiment was repeated. The viability was calculated as follows:

$$\left(\frac{\text{nonstained}\;\text{eggs}}{\text{total}\;\text{eggs}}\right)\times 100$$

### *Solamargine* analysis

A 1200 HPLC 6230 TOF mass spectrometer (Agilent, Santa Clara, CA) was used to determine the concentration of solamargine in each of the 8-wk-old *S. sisymbriifolium* extracts, as described previously (Popova et al., 2022). This was performed using standards of solamargine in a mass spectrometer. These data were the result of three analytical replicates from the same source of plant extracts as the hatch and viability assays. This was not performed for the 4- and 6-wk-old *S. sisymbriifolium* extracts due to their lack of biological activity.

### Data analysis

The RStudio version 2022.07.1 build 554 was used to perform statistical data analysis (RStudio, PBC). To determine whether repeated experiments could be combined for analysis, Pearson’s test was performed between the means of the repeat experiments. There was no statistically significant difference (P > F = 1.0) between the experiments; therefore, the data were combined for further analysis. The hatch and viability reduction percentages were analyzed using ANOVA to determine significance between the extract treatments. The assumptions of normality and homoscedasticity were met. Then, a pairwise comparison was used to assess treatments to the PRD only control with the adjustment method Bonferroni, which is a more conservative pairwise comparison. Zero count values had 0.1 added to them to allow for analysis of the whole dataset. Percentage hatch inhibition over PRD was calculated as follows:

$$\left(\frac{\text{PRD}\;\text{control}\;-\;\text{treatment}}{\text{PRD}\;\text{Control}}\right)\times 100$$

## Results

### The impact of 4-, 6-, or 8-wk-old *S. sisymbriifolium* Extracts on the Hatch and Viability of *G. pallida*

There was no significant difference in the hatch and viability of *G. pallida* when exposed to 4- or 6-wk-old *S. sisymbriifolium* extracts. Hatch from 4-wk-old *S. sisymbriifolium* extracts ranged from 52% to 72% and viability ranged from 78% to 92%. Hatch from 6-wk-old *S. sisymbriifolium* extracts ranged from 45% to 77% and viability ranged from 82% to 97%.

However, when 8-wk-old *S. sisymbriifolium* tissue was used, the extracts significantly reduced the hatch of *G. pallida* depending on the type of extract to which the cysts were exposed. Hatch was inhibited by 68% when *G. pallida* was exposed to the non-diluted 1-butanol extract of the stem/leaf tissue (Tables 1,2). The second highest reduction of hatch (50%) compared with the PRD control was observed in the non-diluted hexane extract of the stem/leaf tissue. Significant effects on hatch were also observed from the 1:2.5 dilution of the stem/leaf tissue from the 1-butanol and hexane fractions (43% and 47%, respectively); and the 1:5 dilution of hexane and 1-butanol stem/leaf fractions (41% and 47%, respectively) (Tables 1,2). There were no significant differences in hatch when *G. pallida* was exposed to a 1:10 dilution of the extracts (Tables 1,2).

**Table 1:** Effect of *S. sisymbriifolium* extracts made from 8-wk-old plant tissues extracted with hexane, dichloromethane, ethyl acetate, or 1-butanol when dissolved in potato root diffusate (PRD) at no dilution or at dilutions of 1:2.5, 1:5, or 1:10 on the hatch of *G. pallida*.

	**Treatment**	**No dilution^z^**	**1:2.5**	**1:5**	**1:10**
Stem/leaf	Hexane	39.1 ± 6.3bd	35.0 ± 7.5bc	40.4 ± 6.2c	63.8 ± 4.9a
	Dichloromethane	64.9 ± 5.6a	68.8 ± 5.2a	60.3 ± 7.4a	62.9 ± 8.2a
	Ethyl acetate	66.2 ± 6.2a	59.9 ± 6.9a	64.6 ± 5.6a	67.4 ± 6.0a
	1-Butanol	24.5 ± 5.7bc	39.1 ± 5.0c	44.8 ± 5.2bc	65.0 ± 6.4a
Root	Hexane	69.0 ± 4.5a	72.1 ± 4.8a	75.2 ± 7.2a	76.3 ± 5.4a
	Dichloromethane	53.1 ± 4.9ad	62.4 ± 5.4a	65.3 ± 5.0ab	70.5 ± 6.4a
	Ethyl acetate	66.9 ± 4.7a	67.4 ± 5.6a	71.8 ± 5.1a	74.6 ± 4.6a
	1-Butanol	76.0 ± 6.4a	82.4 ± 3.4a	66.5 ± 3.8a	81.5 ± 2.6a
PRD	PRD	72.6 ± 5.0a	66.9 ± 5.4a	63.5 ± 4.8a	79.8 ± 3.7a
BSD	BSD	12.1 ± 2.5c	9.8 ± 3.4b	8.6 ± 2.3d	12.6 ± 2.8b

z Data are mean ± standard error (N = 16). The letters represent significance levels, with no significant difference at level *P* < 0.05 for treatments with the same letter. BSD = bare soil diffusate; PRD = potato root diffusate.

**Table 2:** Percentage inhibition of *G. pallida* hatch. PRD control compared with *S. sisymbriifolium* extracts made from 8-wk-old plant tissues extracted with hexane, dichloromethane, ethyl acetate, or 1-butanol when dissolved in potato root diffusate (PRD) at no dilution or at dilutions of 1:2.5, 1:5, or 1:10.

	**Treatment**	**No dilution^z^**	**1:2.5**	**1:5**	**1:10**
Stem/leaf	Hexane	49.5bd	47.3bc	46.6c	20.1a
	Dichloromethane	16.0a	−18.5a	13.6a	21.2a
	Ethyl acetate	14.4a	6.4a	14.5a	15.6a
	1-Butanol	68.3bc	43.0c	40.7bc	18.5a
Root	Hexane	10.7a	−8.3a	0.6a	4.4a
	Dichloromethane	21.9ad	−3.3a	13.7ab	11.7a
	Ethyl acetate	8.5a	−1.3a	−2.7a	6.6a
	1-Butanol	1.7a	−23.8a	5.0a	−2.1a
PRD	PRD	0.0a	0.0a	0.0a	0.0a
BSD	BSD	85.4c	85.2b	89.9d	84.2b

z Data are mean ± standard error (N = 16). The letters represent significance levels, with no significant difference at level *P* > 0.05 for treatments with the same letter. BSD = bare soil diffusate; PRD = potato root diffusate.

The viability of *G. pallida* was significantly reduced by hexane and 1-butanol 8-wk-old *S. sisymbriifolium* plant extracts. The highest inhibition of viability was in the stem/leaf tissue of the undiluted 1-butanol fraction (33%), 1:2.5 dilution (33%), and 1:5 dilution (36%) (Tables 3,4). The second lowest viability compared with the control was the undiluted stem/leaf tissue extract from hexane, with no dilution at 29%, 1:2.5 dilution at 33%, and 1:5 dilution at 20% inhibition (Tables 3,4). None of the extracts significantly reduced the viability of *G. pallida* when diluted to 1:10 (Tables 3,4).

**Table 3:** Effect of *S. sisymbriifolium* extracts made from 8-wk-old plant tissues extracted with hexane, dichloromethane, ethyl acetate, or 1-butanol when dissolved in potato root diffusate (PRD) at no dilution or at dilutions of 1:2.5, 1:5, or 1:10 on the viability of *G. pallida*.

	**Treatment**	**No dilution^z^**	**1:2.5**	**1:5**	**1:10**
Stem/leaf	Hexane	66.7 ± 3.0b	58.3 ± 3.9b	76.1 ± 4.3b	83.4 ± 2.9a
	Dichloromethane	87.8 ± 2.5a	84.7 ± 2.6a	92.8 ± 1.1a	90.9 ± 1.6a
	Ethyl acetate	81.6 ± 4.0a	83.9 ± 2.8a	89.8 ± 2.3a	86.6 ± 2.6a
	1-Butanol	62.1 ± 5.6b	58.3 ± 3.9b	61.2 ± 5.4b	87.9 ± 1.9a
Root	Hexane	84.0 ± 3.0ac	86.9 ± 2.3a	90.3 ± 1.6a	94.1 ± 0.9a
	Dichloromethane	86.6 ± 2.7a	84.7 ± 2.3a	92.3 ± 1.2a	90.2 ± 1.1a
	Ethyl acetate	85.1 ± 2.9a	84.1 ± 2.4a	91.7 ± 1.8a	89.8 ± 1.6a
	1-Butanol	84.0 ± 2.5a	80.9 ± 3.0a	88.7 ± 2.9ab	88.2 ± 1.5a
PRD	PRD	93.3 ± 0.9a	87.2 ± 2.2a	95.1 ± 0.8a	89.5 ± 1.6a
BSD	BSD	88.5 ± 1.7a	90.3 ± 1.2a	89.1 ± 1.4a	90.9 ± 1.8a

z Data are mean ± standard error (N = 16). The letters represent significance levels, with no significant difference at level *P* > 0.05 for treatments with the same letter. BSD = bare soil diffusate; PRD = potato root diffusate.

**Table 4:** Percent inhibition of *G. pallida* viability. PRD control compared with *S. sisymbriifolium* extracts made from 8-wk-old plant tissues extracted with hexane, dichloromethane, ethyl acetate, or 1-butanol when dissolved in potato root diffusate (PRD) at no dilution or at dilutions of 1:2.5, 1:5, or 1:10.

	**Treatment**	**No dilution^z^**	**1:2.5**	**1:5**	**1:10**
Stem/leaf	Hexane	28.5b	33.1b	12.0b	6.8a
	Dichloromethane	5.9a	2.9a	2.4a	−1.5a
	Ethyl acetate	12.5a	3.8a	5.6a	3.3a
	1-Butanol	33.4b	33.1b	35.7b	1.9a
Root	Hexane	9.9ac	0.3a	5.1a	−5.1a
	Dichloromethane	7.1a	2.9a	2.9a	−0.7a
	Ethyl acetate	8.8a	3.5a	3.5a	−0.3a
	1-Butanol	9.9a	7.2a	6.7ab	1.5a
PRD	PRD	0.0a	0.0a	0.0a	0.0a
BSD	BSD	5.1a	−3.6a	6.4a	−1.6a

z Data are mean ± standard error (N = 16). The letters represent significance levels, with no significant difference at level *P* > 0.05 for treatments with the same letter. BSD = bare soil diffusate; PRD = potato root diffusate.

### Concentration of *Solamargine* in 8-wk-old *S. sisymbriifolium* Plants

The 1-butanol extract of stem/leaf tissue of 8-wk-old *S. sisymbriifolium* had the highest concentration of solamargine at 116.9 mg/liter (Table 5). The next highest concentration was in the dichloromethane extract of 8-wk-old stem/leaf tissue at 32.9 mg/liter of solamargine (Table 5).

**Table 5:** Solamargine concentration (mg/liter) found in extracts made from 8-wk-old *S. sisymbriifolium* roots or stem/leaves.

**Plant part**	**Extract**	**Solamargine concentration (mg/liter)^z^**
Stem/leaf	Hexane	28.7 ± 0.2
	Dichloromethane	32.9 ± 0.2
	Ethyl acetate	3.4 ± 0.01
	1-Butanol	116.9 ± 0.8
Root	Hexane	3.7 ± 0.01
	Dichloromethane	4.7 ± 0.02
	Ethyl acetate	2.7 ± 0.002
	1-Butanol	20.4 ± 0.1

z Data are mean ± standard error (N = 3).

## Discussion

This study indicates that extracts made from *S. sisymbriifolium* tissue of a certain age and development are toxic to *G. pallida* in the presence of PRD, which contains a hatching factor essential for occlusion. Pillai and Dandurand (2021) suggested that PRD is essential as it affects egg membrane permeability which then allows large molecules such as glycoalkaloids to penetrate an otherwise impervious eggshell.

The 1-butanol extraction of *S. sisymbriifolium* 8-wk-old leaf and stem tissue is toxic, whereas 4-wk-old and 6-wk-old *S. sisymbriifolium* tissue has no impact on nematode hatch. There could be various reasons why secondary metabolites would be different between the different ages of plants, including differences in environmental factors, genotype, developmental stage, or physiology (Berini et al., 2018; Isah, 2019). This study used plant seed from the same source, as well as controlled environmental factors in greenhouse conditions, thus the variations in toxicity may be due primarily to differences in the developmental stages of the plant. Observed developmental differences in 8-wk-old plants included the development of flowers: 4- and 6-wk-old plants did not show flower development. Additionally, the current study found that compounds present within the hexane extract significantly reduced the hatch and viability of *G. pallida*, which is a previously unreported finding. The reason for the toxicity of these two extracts remains unknown but it is possible that the 1-butanol extract may contain glycoalkaloids, which are known to be toxic to *G. pallida* (Gupta et al., 2014; Pillai and Dandurand, 2021). The hexane extract may contain isoflavones, which are known to be toxic to *Heterodera glycines*, and sterols, which are involved in plant resistance to *Meloidogyne incognita* (Zinovieva et al., 1990; Carter et al., 2018; Cabianca et al., 2021).

Increased solamargine concentration correlates with decreased hatch and viability. Solamargine has been previously shown to be nematicidal (Pillai and Dandurand, 2021) at 100 mg/liter and 200 mg/liter. Results for 4- and 6-wk-old plants are not shown but 8-wk-old plants are at the flowering stage, which correlates with a change in secondary metabolites in plants (Berini et al., 2018; Isah, 2019).

All toxic effects of the secondary metabolites of *S. sisymbriifolium* with the hexane and 1-butanol extracts on *G. pallida* were found to be concentration dependent, which is in agreement with previous studies on secondary metabolite toxicity (Cantelo et al., 1987; Sanford et al., 1995; Sanford et al., 1996; Yencho et al., 2000). The mortality of potato leafhopper was found to be significantly higher at the highest concentration of glycoalkaloid and decreased with decreasing glycoalkaloid concentration (Sanford et al., 1996). Resistance to Colorado potato beetle correlates with an increase in concentration of the leptine class of glycoalkaloids, which are found in plants (Yencho et al., 2000). Significant reductions in the hatch and viability of *G. pallida* were found in the current study at undiluted concentrations of extract to a 1:5 dilution, but not at a 1:10 dilution. The largest reduction in hatch percentage was 68% compared with the PRD control in the undiluted 1-butanol stem/ leaf extract. In this study, the greatest impact found on hatch was from extracts that were not diluted.

The 1-butanol extract from *S. sisymbriifolium* potentially contains anthocyanins, glucosides, glycosides, glycoalkaloids, and steroidal saponins, which could be toxic to *G. pallida* (Dinan et al., 2001; Sánchez-Maldonado et al., 2014). Anthocyanins contained in *Pinus pinaster* increased when infected with the pinewood nematode *Bursaphelenchus xylophilus*, indicating a plant defense response (Nunes da Silva et al., 2021). Glucosides and glycosides are another potential source of toxicity to *G. pallida* that would be found in the 1-butanol extract. It has been reported that glycosides release nematicidal compounds that are toxic to *Meloidogyne hapla*, as well as other nematodes (Chitwood, 2002). Glycoalkaloids are the chemicals in *S. sisymbriifolium* that may be responsible for the toxic impact on *G. pallida*. It is reported that pure glycoalkaloids reduce the hatch of *G. pallida* by 99% depending on concentration (Pillai and Dandurand, 2021). Steroidal saponins are another potential source of toxins in *S. sisymbriifolium* stem/leaf tissue 1-butanol extract. Saponins have been shown to be toxic to *Panagrellus redivivus* (Chitwood, 2002).

As hexane is a non-polar solvent, the hexane extract from *S. sisymbriifolium* will contain different chemicals than the 1-butanol extract and potentially contains isoflavones and sterols, which are both toxic to various nematodes (Dinan et al., 2001). However, isoflavones are found exclusively in legumes and appear to play a role in protecting soybeans from the soybean cyst nematode *Heterodera glycines* (Carter et al., 2018). As isoflavones are only present in legumes, it is unlikely that isoflavones will be found in *S. sisymbriifolium*. Non-polar sterols have shown concentration-dependent toxicity to various nematodes (Cabianca et al., 2021). Plant sterols in tomato are involved in resistance to *Meloidogyne incognita* (Zinovieva et al., 1990). Owing to *S. sisymbriifolium* being in the same family (Solanaceae) as tomato, the sterols present in these plants may be a promising source of compounds that are toxic to plant parasitic nematodes, such as *G. pallida*.

Many synthetic nematicides have been banned due to environmental concerns, making this study important for the development of an environmentally friendly and sustainable nematicide for use against potato cyst and other nematodes. This study indicates that there may be more than one chemical in *S. sisymbriifolium* toxic to *G. pallida.* Future studies on the identification of the secondary metabolites found within the hexanes and 1-butanol extracts of *S. sisymbriifolium* are essential for understanding which compounds have an impact on nematodes such as *G. pallida*. Fractionation of these extracts via HPLC, and then testing the fractions against *G. pallida*, would enable increased understanding of the impact these metabolites from *S. sisymbriifolium* may have. More studies on *S. sisymbriifolium* secondary metabolites and their effect on *G. pallida* are needed.

## References

[j_jofnem-2024-0027_ref_001] Alzérreca A., Hart G. (1982). Molluscicidal steroid glycoalkaloids possessing stereoisomeric spirosolane structures. Toxicology Letters.

[j_jofnem-2024-0027_ref_002] Bekkouche K., Markouk M., Larhsini M., Jana M., Lazrek H. B. (2000). Molluscicidal properties of glycoalkaloid extracts from Moroccan *Solanum* species. Phytotherapy Research.

[j_jofnem-2024-0027_ref_003] Berini J., Brockman S., Hegeman A., Reich P., Muthukrishnan R., Montgomery R., Forester J. (2018). Combinations of abiotic factors differentially alter production of plant secondary metabolites in five woody plant species in the boreal-temperate transition zone. Frontiers in Plant Science.

[j_jofnem-2024-0027_ref_004] CABI (2018). *Globodera pallida* (white potato cyst nematode).

[j_jofnem-2024-0027_ref_005] Cabianca A., Müller L., Pawlowski K., Dahlin P. (2021). Changes in the plant β-sitosterol/stigmasterol ratio caused by the plant parasitic nematode *Meloidogyne incognita*. Plants.

[j_jofnem-2024-0027_ref_006] Cantelo W., Douglass L., Sanford L., Sinden S., Deahl K. (1987). Measuring resistance to the Colorado potato beetle (Coleoptera, *Chrysomelidae*) in potato. Journal Entomological Science.

[j_jofnem-2024-0027_ref_007] Carter A., Tenuta A., Rajcan I., Welacky T., Woodrow L., Eskandari M. (2018). Identification of quantitative trait loci for seed isoflavone concentration in soybean (*Glycine max*) against soybean cyst nematode stress. Plant Breeding.

[j_jofnem-2024-0027_ref_008] Chauhan K., Sheth N., Ranpariya V., Parmar S. (2011). Anticonvulsant activity of solasodine isolated from *Solanum sisymbriifolium* fruits in rodents. Pharmaceutical Biology.

[j_jofnem-2024-0027_ref_009] Chitwood D. J. (2002). Phytochemical based strategies for nematode control. Annual Review of Phytopathology.

[j_jofnem-2024-0027_ref_010] Contina J., Dandurand L. M., Knudsen G. (2019). A predictive risk model analysis of the potato cyst nematode *Globodera pallida* in Idaho. Plant Disease.

[j_jofnem-2024-0027_ref_011] Dandurand L.M., Knudsen G. (2016). Effect of the trap crop *Solanum sisymbriifolium* and two biocontrol fungi on reproduction of the potato cyst nematode, *Globodera pallida*. Annals of Applied Biology.

[j_jofnem-2024-0027_ref_012] Dinan L., Harmatha J., Lafont R. (2001). Chromatographic procedures for the isolation of plant steroids. Journal of Chromatography A..

[j_jofnem-2024-0027_ref_013] Ferro E. A., Alvarenga N. L., Ibarrola D. A., Hellión-Ibarrola M. C., Ravelo A. G. (2005). A new steroidal saponin from *Solanum sisymbriifolium* roots. Fitoterapia.

[j_jofnem-2024-0027_ref_014] Gajger I. T., Dar S. A. (2021). Plant allelochemicals as sources of insecticides. Insects.

[j_jofnem-2024-0027_ref_015] Gupta V. K., Simlai A., Tiwari M., Bhattacharya K., Roy A. (2014). Phytochemical contents, antimicrobial and antioxidative activities of *Solanum sisymbriifolium*. Journal of Applied Pharmaceutical Science.

[j_jofnem-2024-0027_ref_016] Hafez S. L., Sundararaj P., Handoo Z. A., Skantar L., Carta K., Chitwood D. J. (2007). First report of the pale cyst nematode, *Globodera pallida*, in the United States. Plant Disease.

[j_jofnem-2024-0027_ref_017] Hartmann T., Rosenthal G. A., Berenbaum M. R. (1991). Herbivores, their interactions with secondary plant metabolites. The Chemical Participants.

[j_jofnem-2024-0027_ref_018] Ibarrola D., Hellion-Ibarrola M., Alvarenga N., Ferro E., Hatakeyama N., Shibuya N., Yamazaki M., Momose Y., Yamamura S., Tsuchida K. (2006). Cardiovascular action of nuatigenosido from *Solanum sisymbriifolium*. Pharmaceutical Biology.

[j_jofnem-2024-0027_ref_019] Isah T. (2019). Stress and defense responses in plant secondary metabolites production. Biological Research.

[j_jofnem-2024-0027_ref_020] Koirala S., Watson P., McIntosh C. S., Dandurand L. M. (2020). Economic impact of *Globodera pallida* on the Idaho economy. American Journal of Potato Research.

[j_jofnem-2024-0027_ref_021] Kooliyottil R., Dandurand L. M., Govindan B. N., R Knudsen G. (2016). Microscopy method to compare cyst nematode infection of different plant species. Advances in Bioscience and Biotechnology.

[j_jofnem-2024-0027_ref_022] Mazzola P. G., Lopes A. M., Hasmann F. A., Jozala A. F., Penna T. C. V., Magalhaes P. O., Rangel-Yagui C. O., Pessoa A. (2008). Liquid-liquid extraction of biomolecules: An overview and update of the main techniques. Journal of Chemical Technology and Biotechnology.

[j_jofnem-2024-0027_ref_023] More G. K. (2019). A review of the ethnopharmacology, phytochemistry and pharmacological relevance of the South African weed *Solanum sisymbriifolium Lam.(Solanaceae)*. Environment, Development and Sustainability.

[j_jofnem-2024-0027_ref_024] Nour S. M., Lawrence J. R., Zhu H., Swerhone G. D., Welsh M., Welacky T. W., Topp E. (2003). Bacteria associated with cysts of the soybean cyst nematode (*Heterodera glycines*). Applied and Environmental Microbiology.

[j_jofnem-2024-0027_ref_025] Nunes da Silva M., Santos C. S., Cruz A., López-Villamor A., Vasconcelos M. W. (2021). Chitosan increases *Pinus pinaster* tolerance to the pinewood nematode (*Bursaphelenchus xylophilus*) by promoting plant antioxidative metabolism. Scientific Reports.

[j_jofnem-2024-0027_ref_026] Pillai S. S., Dandurand L. M. (2019). Evaluation of fluorescent stains for viability assessment of the potato cyst nematodes *Globodera pallida* and *Globodera ellingtonae*. Advances in Bioscience and Biotechnology.

[j_jofnem-2024-0027_ref_027] Pillai S. S., Dandurand L. M. (2021). Effect of steroidal glycoalkaloids on hatch and reproduction of potato cyst nematode, *Globodera pallida*. Plant Disease.

[j_jofnem-2024-0027_ref_028] Popova I., Sell B., Pillai S. S., Kuhl J., Dandurand L. M. (2022). High-performance liquid chromatography–mass spectrometry analysis of glycoalkaloids from underexploited *Solanum* species and their acetylcholinesterase inhibition activity. Plants.

[j_jofnem-2024-0027_ref_029] Sánchez-Maldonado A. F., Mudge E., Gänzle M. G., Schieber A. (2014). Extraction and fractionation of phenolic acids and glycoalkaloids from potato peels using acidified water/ethanol-based solvents. Food Research International.

[j_jofnem-2024-0027_ref_030] Sanford L. L., Deahl K. L., Sinden S. L., Kobayashi R. S. (1995). Glycoalkaloid content in tubers of hybrid and backcross populations from a *Solanum tuberosumx s. chacoense* cross. American Potato Journal.

[j_jofnem-2024-0027_ref_031] Sanford L. L., Domek J. M., Cantelo W. W., Kobayashi R. S., Sinden S. L. (1996). Mortality of potato leafhopper adults on synthetic diets containing seven glycoalkaloids synthesized in the foliage of various *Solanum* species. American Potato Journal.

[j_jofnem-2024-0027_ref_032] Schäfer H., Wink M. (2009). Medicinally important secondary metabolites in recombinant microorganisms or plants: Progress in alkaloid biosynthesis. Biotechnology Journal.

[j_jofnem-2024-0027_ref_033] Scholte K. (2000a). Growth and development of plants with potential for use as trap crops for potato cyst nematodes and their effects on the numbers of juveniles in cysts. Annals of Applied Biology.

[j_jofnem-2024-0027_ref_034] Scholte K. (2000b). Screening of non-tuber bearing *Solanaceae* for resistance to and induction of juvenile hatch of potato cyst nematodes and their potential for trap cropping. Annals of Applied Biology.

[j_jofnem-2024-0027_ref_035] Scholte K., Vos J. (2000). Effects of potential trap crops and planting date on soil infestation with potato cyst nematodes and root-knot nematodes. Annals of Applied Biology.

[j_jofnem-2024-0027_ref_036] Skantar A. M., Handoo Z. A., Carta L. K., Chitwood D. J. (2007). Morphological and molecular identification of *Globodera pallida* associated with potato in Idaho. Journal of Nematology.

[j_jofnem-2024-0027_ref_037] Treutter D. (2005). Significance of flavonoids in plant resistance and enhancement of their biosynthesis. Plant Biology.

[j_jofnem-2024-0027_ref_038] Tzanova M., Atanasov V., Yaneva Z., Ivanova D., Dinev T. (2020). Selectivity of current extraction techniques for flavonoids from plant materials. Processes.

[j_jofnem-2024-0027_ref_039] USDA - APHIS (2023). Pale cyst nematode (PCN) eradication program - Idaho Falls, Idaho 2023 1st quarter report.

[j_jofnem-2024-0027_ref_040] USDA-APHIS (2009). Potato cyst nematode national survey and diagnostic cyst sample forwarding protocols.

[j_jofnem-2024-0027_ref_041] Yencho C. G., Kowalski S. P., Kennedy G. G., Sanford L. L. (2000). Segregation of leptine glycoalkaloids and resistance to Colorado potato beetle (*Leptinotarsa decemlineata* (Say)) in F2 *Solanum tuberosum* (4x) x *S. chacoense* (4x) potato progenies. American Journal of Potato Research.

[j_jofnem-2024-0027_ref_042] Zinovieva S. V., Vasyukova N. I., Ozeretskovaskaya I. L. (1990). Involvement of plant sterols in the system tomatoes - nematode *Meloidogyne incognita*. Helminthologia.

